# Supplementary material for: Inhibitory role of Annexin A1 in pathological bone resorption and therapeutic implications in periprosthetic osteolysis
Source: Nat Commun. 2022 Jul 7;13:3919. doi: 10.1038/s41467-022-31646-0 (PMC9262976; doi:10.1038/s41467-022-31646-0)

Supplemental Information

Inhibitory role of annexin A1 in pathological bone resorption and therapeutic implications in periprosthetic osteolysis

Hend Alhasan[1], Mohamad Alaa Terkawi[1*], Gen Matsumae[1], Taku Ebata[1], Yuan Tian[1], Tomohiro Shimizu[1*], Yoshio Nishida[1], Shunichi Yokota[1], Fayna Garcia-Martin[2,3], Mahmoud M. Abd Elwakil[4], Daisuke Takahashi[1], Mahmoud A. Younis[4], Hideyoshi Harashima[4], Ken Kadoya[1], Norimasa Iwasaki[1]

[1]Department of Orthopedic Surgery, Faculty of Medicine and Graduate School of Medicine, Hokkaido University, Kita-15, Nish-7, Kita-ku, Sapporo, 060-8638, Japan.

[2]Graduate School and Faculty of Advanced Life Science, Laboratory of Advanced Chemical Biology, Hokkaido University, N21 W11, Kita-21, Nish-11, Kita-ku, Sapporo 001-0021, Japan.

[3]Faculty of Science and Technology, Department of Chemistry, University of La Rioja, E-26006 Logroño, Spain.

[4]Laboratory of Innovative Nanomedicine, Faculty of Pharmacy and Pharmaceutical Sciences, Hokkaido University, Kita-12 Nishi-6, Kita-Ku Sapporo 060-0812, Japan.

[*]Correspondence

M A Terkawi, materkawi@med.hokudai.ac.jp

T Shimizu, simitom@wg8.so-net.ne.jp

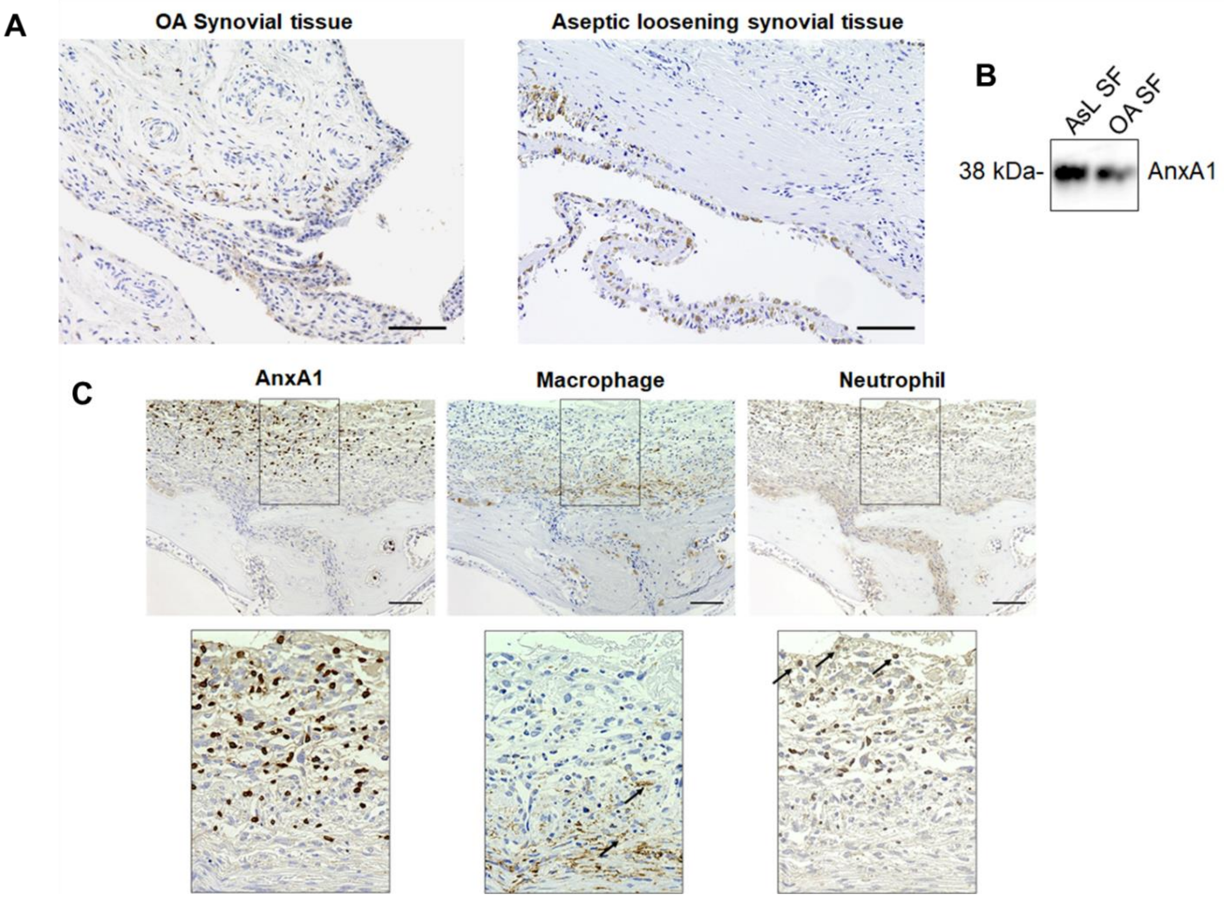

Supplementary Figure 1. Detection of AnxA1 in periprosthetic tissues of clinical and experimental model samples. A) IHC staining showing AnxA1 expression in synovial hip tissues collected from patients diagnosed OA and these who are undergoing revision surgery due to aseptic loosening. B) AnxA1 expression in synovial fluids of the same patient as detected by Western blot analysis. C) Detection of AnxA1 in in tissues formed around UHMWPE debris in mouse model by IHC staining with specific antibodies targeting AnxA1, CD68 macrophages and neutrophil elastase. Arrows indicate the $CD68^{+}AnxA1^{+}$ (left panel) or Elastase $^{+}AnxA1^{+}$ (right panel) cells. Scale bars are 100 µm. Staining experiments were repeated at least twice with similar results for reproducibility of data. Uncropped blot is provided in Supplementary Figure 12.

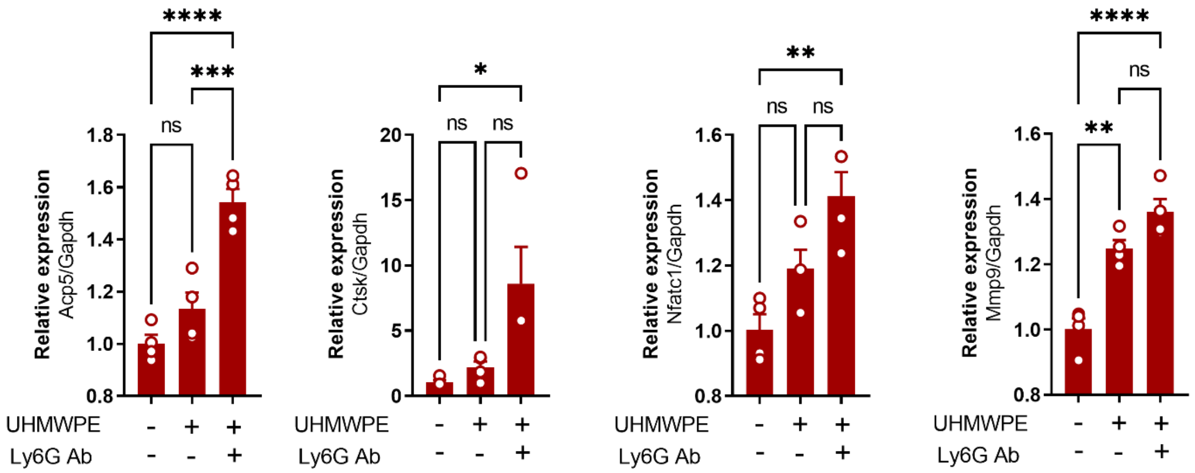

Supplementary Fig. 2. Gene expression of the major osteoclast markers in calvarial bone tissue. Results represent the means of relative expression values ± SEM from 4 mice (n = 4 ± SEM). Significant difference between the groups was determined by one-way ANOVA, followed by Tukey's multiple-comparison procedure. * p < 0.05, ** p < 0.001, *** p < 0.0001, **** p< 0.00001. ns, not significant. Experiments were repeated at least three time for reproducibility of data. Source data are provided as a Source Data file.

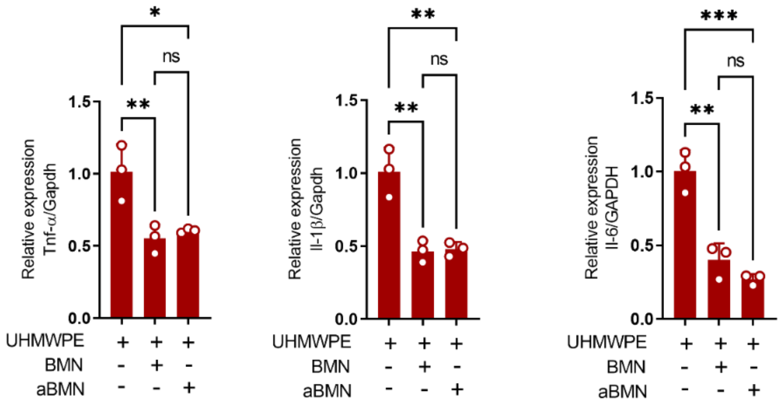

Supplementary Fig. 3. Gene expression of inflammatory molecules in granulomatous tissue using qRT-PCR. Adoptive transfer of neutrophil suppressed local inflammation triggered by implantation of UHMWPE debris. Results represent the means of relative expression values ± SEM from 3 mice (n = 3 ± SEM). Significant difference between the groups was determined by one-way ANOVA, followed by Tukey's multiple-comparison procedure. * p < 0.05, ** p < 0.001, *** p < 0.0001. ns, not significant. Experiments were repeated at least three time for reproducibility of data. Source data are provided as a Source Data file.

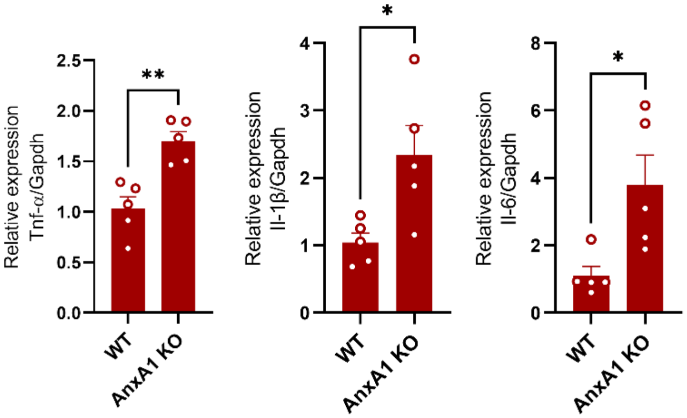

Supplementary Figure 4. Gene expression of inflammatory molecules in granulomatous tissue using qRT-PCR. Tissues were collected from wild type (WT) and AnxA1-defeicint mice (AnxA1 KO) after 7 days of UHMWPE debris implantation. Results represent the means of relative expression values ± SEM from 5 mice (n = 5 ± SEM). Significant difference between the groups was determined by Two-tailed student t-test. * p < 0.05, ** p < 0.001. ns, not significant. Experiments were repeated at least three time for reproducibility of data. Source data are provided as a Source Data file.

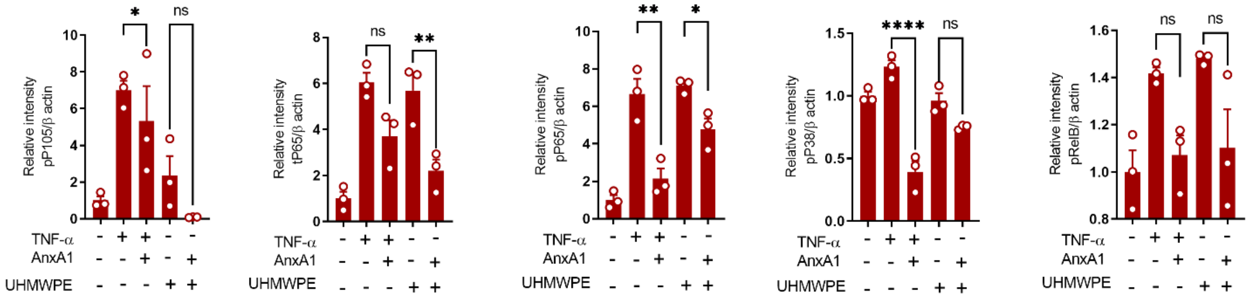

Supplementary Figure 5. Quantification of relative intensity of bands detected by Western blotting. Detected bands for each target were subjected to ImageJ for quantification of intensities. Significant difference between the groups was determined by one-way ANOVA, followed by Tukey's multiple-comparison procedure. Results represent relative intensity of bands ± SEM from 3 samples. Significance presents * p < 0.05, * p < 0.05, ** p < 0.001, *** p < 0.0001. Analysis was repeated at least three time for reproducibility of data.

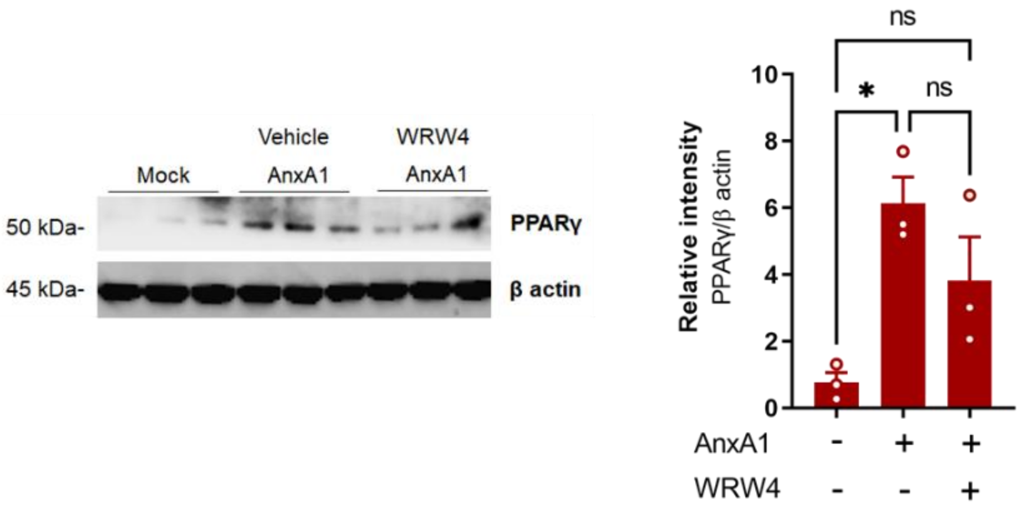

Supplementary Figure 6. Effects of WRW4 treatment on the expression of PPAR-γ in macrophages stimulated with AnxA1. Human macrophages were pretreated with WRW4 (1μM) for 30 min before stimulation with AnxA1. Cells were then washed with PBS and stimulated with recombinant 100 ng/ml AnxA1 for 3 h. Cells were next lysed and subjected to SDS-PAGE and Western blot analysis. Lefts panel is the quantification of relative intensity of bands detected by Western blotting. Results represent relative intensity of bands ± SEM from 3 samples. Significant difference between the groups was determined by one-way ANOVA, followed by Tukey's multiple-comparison procedure. * $p < 0.05$. ns, not significant. Analysis was repeated at least three time for reproducibility of data. Uncropped blots are provided in Supplementary Figure 12.

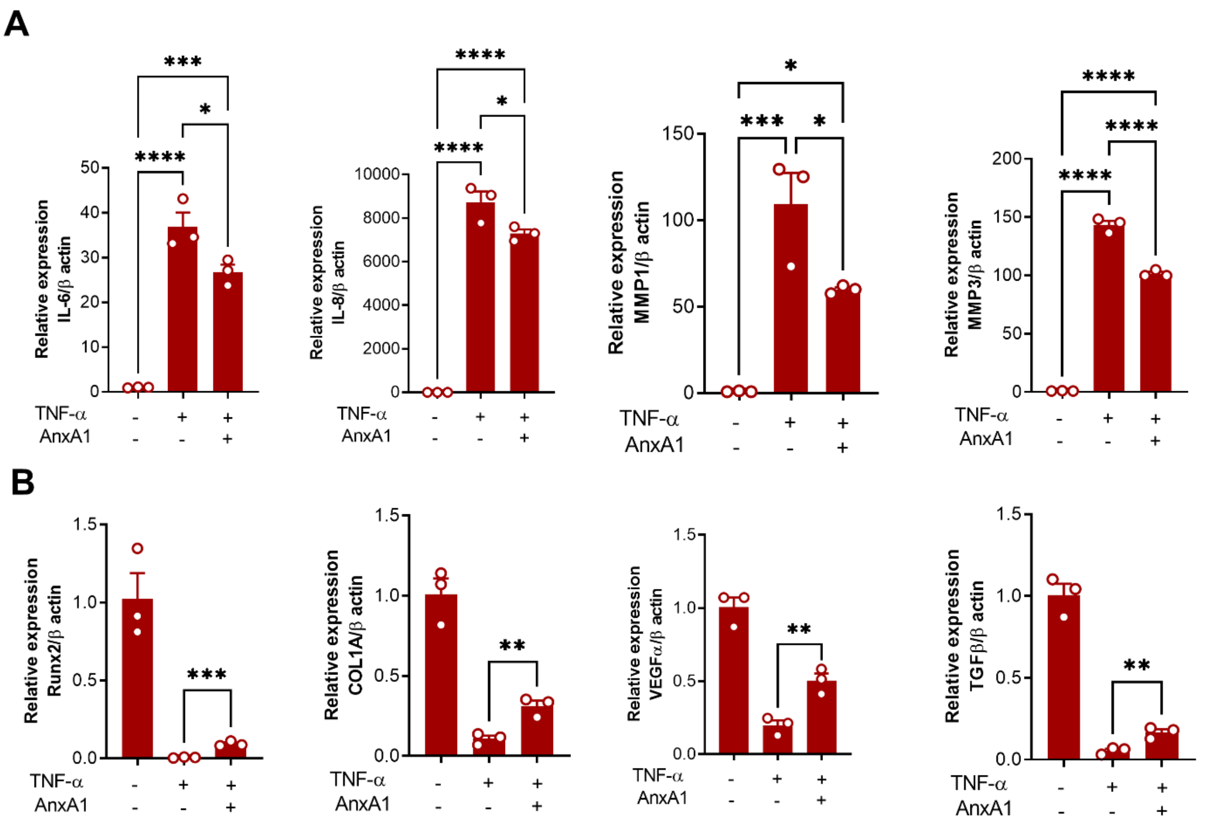

Supplementary Figure 7. Effects of recombinant AnxA1 on the stimulated-FLS and osteoblasts. AnxA1 inhibits the expression of inflammatory cytokines and bone anabolic factors in human FLS (A) and osteoblasts (B) stimulated by TNF-α. Results represent the means of relative expression values ± SEM from triplicates. Significant difference between the groups was determined by one-way ANOVA, followed by Tukey's multiple-comparison procedure. * $p < 0.05$, ** $p < 0.001$, *** $p < 0.0001$, **** $p < 0.00001$. Experiments were repeated at least twice with two different investigators for reproducibility of data.

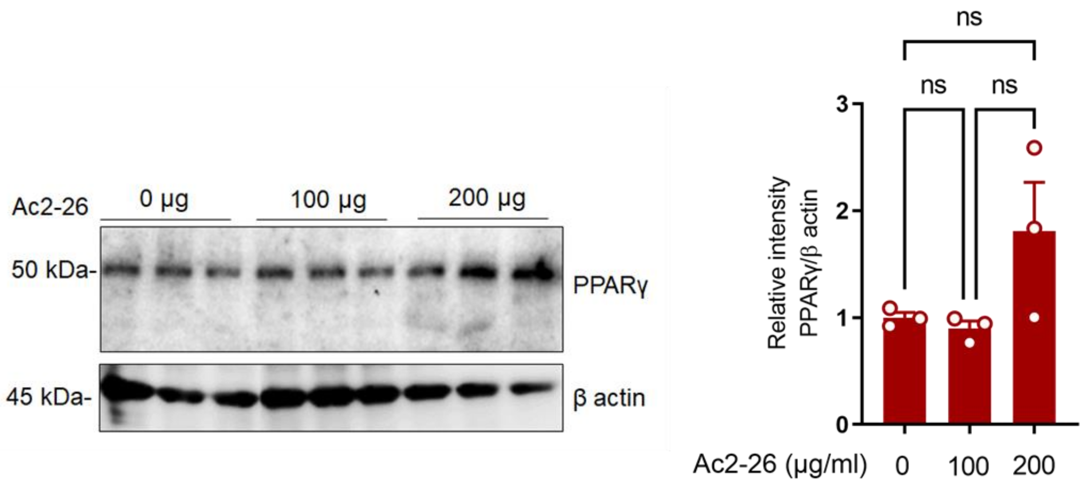

Supplementary Figure 8. Expression of PPAR-γ in THP1 macrophage cell line after stimulation with Ac2-26 peptide. Cells were stimulated for 3h and the expression was analyzed using Western blotting. Right panel shows quantification of relative intensity of bands detected by Western blotting. An increase in the expression of PPAR-γ was observed at 200 µg/ml concentration of peptide. Results represent relative intensity of bands ± SEM from 3 samples. Significant difference between the groups was determined by one-way ANOVA, followed by Tukey's multiple-comparison procedure. Analysis was repeated at least three time for reproducibility of data. ns, not significant. Uncropped blots are provided in Supplementary Figure 12.

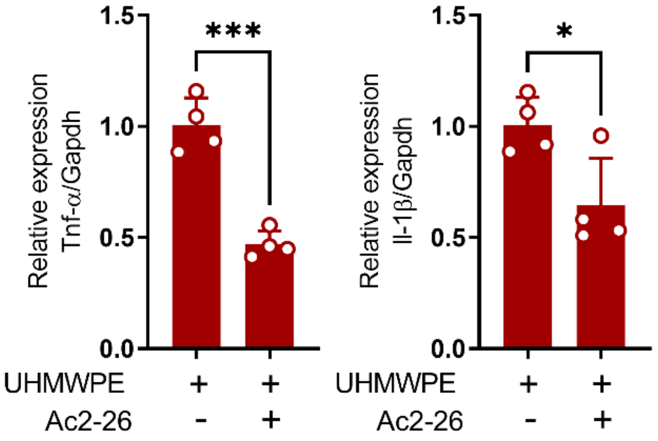

Supplementary Figure 9. Gene expression of major inflammatory cytokines in tissues formed around wear debris on calvarial bone of mice. Results represent the means of relative expression values ± SEM from 4 mice (n = 4 ± SEM). Significant difference between the groups was determined by Two-tailed student t-test. * $p < 0.05$, *** $p < 0.0001$. Analysis was repeated at least three time for reproducibility of data. ns, not significant.

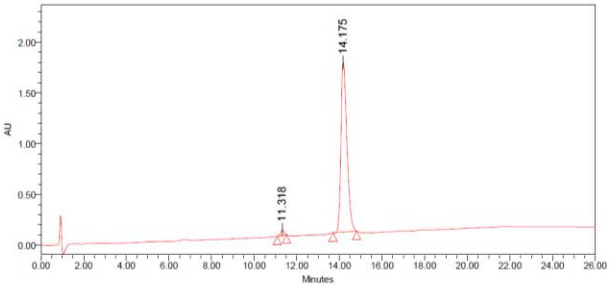

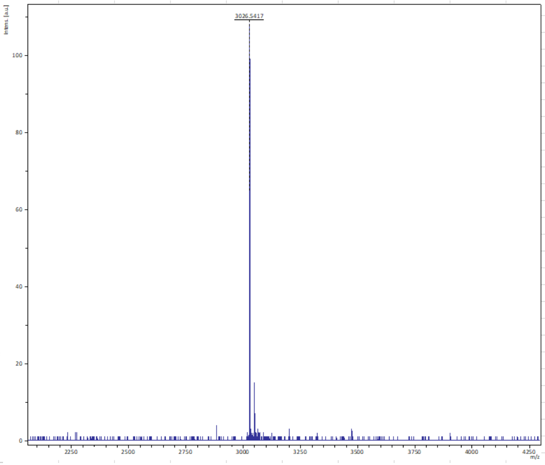

Peak Results

	Name	RT	Height	Area	% Area	Total Area
1		11.318	55995	625034	1.89	33049273
2		14.175	1677298	32424239	98.11	33049273

Supplementary Figure 10. RP-UPLC chromatogram and MALDI-TOF MS spectrum of synthetic Ac2-26 peptide derived from mouse AnxA1. Peptide Ac2-26 (Ac-AMVSEFLKQARFLENQEQEYVQAVK-NH_2) was synthesized by solid-phase peptide synthesis, purified by semipreparative reverse phase high pressure liquid chromatography (RP-HPLC), and then characterized by MALDI-TOF MS spectrometry and RP-UPLC chromatography. The peptide was obtained as a white amorphous solid (13.45 mg), which was detected by RP-UPLC (t_R 14.175 min, purity 98.11% in the linear gradient from 2% to 60% of CH_3CN (0.1% TFA) in H_2O (0.1% TFA) for 20 min). The calculated m/z was noted as 3026.5417 $[M+H]^+$.

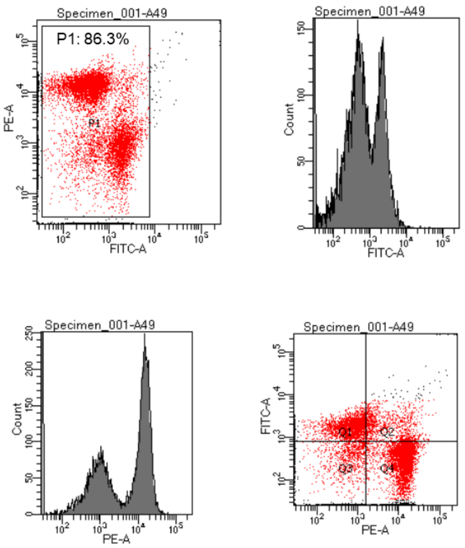

Supplementary Figure 11. Gating strategy of flow cytometric analysis.

Supplementary Table 1. Primers used for genotyping.

Target region	Sequence
Anxa1 LF	5′-TGTATCCTCGGATGTTGCTG-3′
Anxa1LR	5′-GTAATGGGCTCACGGTGTTT-3′
Anxa1MF	5′-ACAGACGCTCAGTTTGCTCA-3′
Anxa1MR	5′-AACACAAAGCTGCCACATCC-3′

Supplementary Table 2. Primers used for gene expression by qRT-PCR.

Target Genes	Forward	Reverse
B actin	CCTCACCCTGAAGTACCCCA	TCGTCCCAGTTGGTGACGAT
hIL-10	GTGTCCGTGAGGTTGGAGGT	AAGGGTTACTTGGGTTGCCA
hTGFβ	CCTGGGGCATCACTTCTACC	TGGAGCTGGTGAAACGGAAG
hAnxA1	TGCACAGCGTCAACAGATCA	CAGTGTTTCATCCAGGGGCT
hRUNX2	TCTCCAGGAGGACAGCAAGA	GCAGCCTTAAATGACTCTGTTGG
hCOL1A	ACTGGCGAAACCTGTATCCG	CCAGTTCTTGGCTGGGATGT
hIL-6	AGAAAAAGGTGGGTGTGTCCT	GTCTTTGAGCCTGTCTTCCCC
hIL-8	TCCAAACCTTTCCACCCCAAA	AATTTCTGTGTTGGCGCAGTG
hVEGFA	AAAACACAGACTCGCGTTGC	CCTCGGCTTGTCACATCTGC
hPPARγ	GGCTACACTGTTCTGCGGAT	CACCCAGATCACAAGCCCAT
mGAPDH	TGCAGCGAACTTTATTGATG	ACTTTGTCAAGCTCATTTCC
mIL-6	TAGTCCTTCCTACCCCAATTTCC	TTGGTCCTTAGCCACTCCTTC
mIL-10	AAGGGTTACTTGGGTTGCCA	CCTGGGGCATCACTTCTACC
mTGF-β	TGGAGCTGGTGAAACGGAAG	CTGGCGAGCCTTAGTTTGGA
mTRAP	TCTTCAGGACGAGAACGGTG	CCTTTCGTTGATGTCGCACA
mCTSK	CAGAAGCAGTATAACAGCAAGG	CCCAAATTAAACGCCGAGAG
mAnxa1	AACCATCGTGAAGTGTGCCA	CTTCGTACAGCTTCTCGGCA
mPPARγ	TGTCTCACAATGCCATCAGGT	CTGGGTTCAGCTGGTCGATA

Supplementary Table 3. Kinetic analysis of the release of the peptide from the optimum hydrogel at 37 °C and pH 7.4. The average cumulative percentage of the peptide dose released at different time points for three independent experiments was used for the calculation.

Correlation coefficient (r)			Assigned equation	$t_{50\%}$[a] (hr)	K_{rel}[b]
Zero-order model	First-order model	Higuchi-diffusion model			
0.9962	-0.1460	0.9451	$M_t / M_\infty = 0.6057\ t$	82.54	0.6057

[a]$t_{50\%}$: the time required for the release of 50% of the peptide dose.

[b]$K_{rel.}$: release rate constant calculated based on the best-fit kinetic model.

Supplementary Table 4. Analysis of the peptide release data from the optimum hydrogel at 37 °C and pH 7.4 according to the Ritger and Peppas equation. The average cumulative percentage of the peptide dose released at different time points for three independent experiments was used for the calculation.

n[a]	K[a]	r[b]	Mechanism
1.2426	0.1789	0.9981	Super case-II transport

[a]n and K are the parameters of Ritger and Peppas equation defined in the methods section (Equation 5).

[b]r is the correlation coefficient calculated by linear regression analysis of the Logarithm of cumulative percentage of drug released (Log M_t / M_∞) versus the Logarithm of the corresponding different time points (Log t).

Supplementary Figure 12. Uncropped blots in Supplementary Figures 1, 6 and 8.

Uncropped blot in Supplementary Figure 1b.

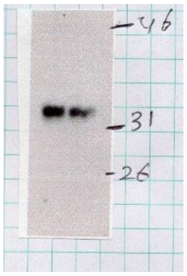

Uncropped blots in Supplementary Figure 6.

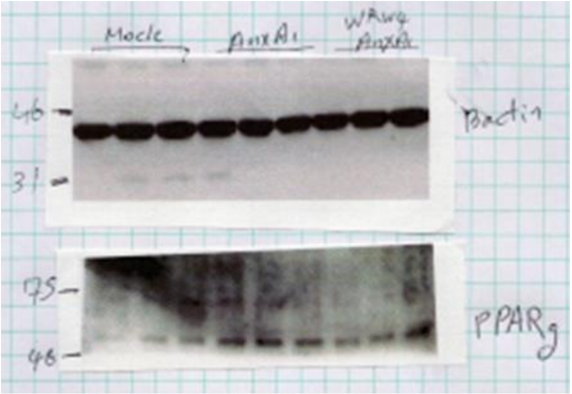

Uncropped blots in Supplementary Figure 8.

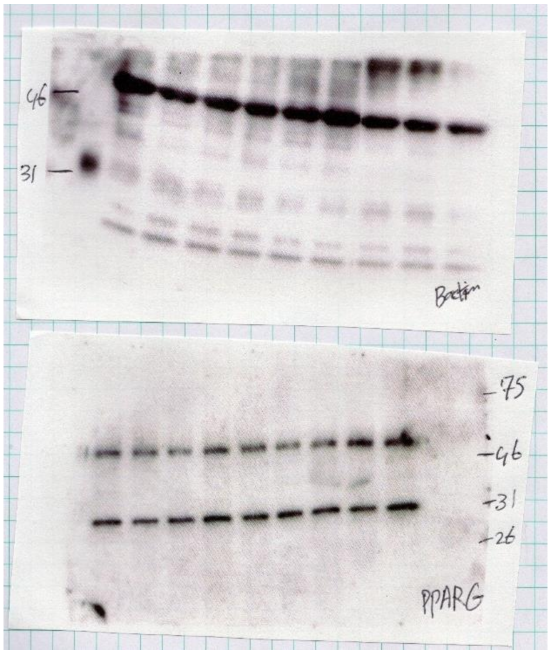

Supplement: Supplementary file 1 — Supplementary Information [file 41467_2022_31646_MOESM1_ESM.pdf]
