# Supplementary material for: Inhibitory role of Annexin A1 in pathological bone resorption and therapeutic implications in periprosthetic osteolysis
Source: Nat Commun. 2022 Jul 7;13:3919. doi: 10.1038/s41467-022-31646-0 (PMC9262976; doi:10.1038/s41467-022-31646-0)
Supplement: Supplementary file 3 — Reporting Summary [file 41467_2022_31646_MOESM3_ESM.pdf]

nature portfolio

Corresponding author(s): M Alaa Terkawi

Last updated by author(s): Apr 5, 2022

Reporting Summary

Nature Portfolio wishes to improve the reproducibility of the work that we publish. This form provides structure for consistency and transparency in reporting. For further information on Nature Portfolio policies, see our Editorial Policies and the Editorial Policy Checklist.

Statistics

For all statistical analyses, confirm that the following items are present in the figure legend, table legend, main text, or Methods section.

n/a	Confirmed	
☐	☒	The exact sample size (*n*) for each experimental group/condition, given as a discrete number and unit of measurement
☐	☒	A statement on whether measurements were taken from distinct samples or whether the same sample was measured repeatedly
☐	☒	The statistical test(s) used AND whether they are one- or two-sided<br>*Only common tests should be described solely by name; describe more complex techniques in the Methods section.*
☐	☒	A description of all covariates tested
☐	☒	A description of any assumptions or corrections, such as tests of normality and adjustment for multiple comparisons
☐	☒	A full description of the statistical parameters including central tendency (e.g. means) or other basic estimates (e.g. regression coefficient) AND variation (e.g. standard deviation) or associated estimates of uncertainty (e.g. confidence intervals)
☐	☒	For null hypothesis testing, the test statistic (e.g. *F*, *t*, *r*) with confidence intervals, effect sizes, degrees of freedom and *P* value noted<br>*Give P values as exact values whenever suitable.*
☒	☐	For Bayesian analysis, information on the choice of priors and Markov chain Monte Carlo settings
☒	☐	For hierarchical and complex designs, identification of the appropriate level for tests and full reporting of outcomes
☒	☐	Estimates of effect sizes (e.g. Cohen's *d*, Pearson's *r*), indicating how they were calculated

Our web collection on statistics for biologists contains articles on many of the points above.

Software and code

Policy information about availability of computer code

Data collection	Hitachi H-7100 transmission electron microscope at 80 kV (JEM-1400, Plus Nihon Denshi, Tokyo, Japan) for TEM images<br>Thermal Cycler Dice Real Time System, Software Ver. 5.00D for TP900 (Takara, Japan)<br>Bz-X Viewer keyence for microscopic imaging<br>Rigaku software (Version 2.2.94.0) for micro-CT images<br>Quantity One v. 4.6.9 (Bio-Rad) software for bands visualization<br>BD FACSDiva software (Version 8.0.1) for flow cytometry analysis
Data analysis	Statistical analyses were performed using GraphPad Software Inc., Prism 9 (Version 9.3.0) La Jolla, CA, USA.<br>STAR software for mapping the genes and RSEM software for read count of the genes and the significant differences were calculated using DESeq2 R package (https://www.r-project.org/).<br>GO analyses were performed using the Database for Annotation Visualization and Integrated Discovery online tools (DAVID: david.abcc.ncifcrf.gov).<br>Heat map was used to visualize the differences in fold changes in each enriched GO term (http://biit.cs.ut.ee/clustvis/).<br>Image J software (National Institutes of Health, NIH, Washington, DC, USA) for quantification and image analysis<br>qRT-PCR data was analyzed using Microsoft 365 Excel version 2204

For manuscripts utilizing custom algorithms or software that are central to the research but not yet described in published literature, software must be made available to editors and reviewers. We strongly encourage code deposition in a community repository (e.g. GitHub). See the Nature Portfolio guidelines for submitting code & software for further information.

Data

Policy information about availability of data

All manuscripts must include a data availability statement. This statement should provide the following information, where applicable:

- Accession codes, unique identifiers, or web links for publicly available datasets
- A description of any restrictions on data availability
- For clinical datasets or third party data, please ensure that the statement adheres to our policy

TThe bulk RNA-seq data in this paper were deposited on NCBI Gene Expression Omnibus (GEO) and are accessible through GEO Series accession numbers; GSE183145 and GSE171542. The remaining data are available within the article, the Supplementary Information or Source Data file provided with this paper. Source data are provided with this paper.

Field-specific reporting

Please select the one below that is the best fit for your research. If you are not sure, read the appropriate sections before making your selection.

[x] Life sciences [ ] Behavioural & social sciences [ ] Ecological, evolutionary & environmental sciences

For a reference copy of the document with all sections, see nature.com/documents/nr-reporting-summary-flat.pdf

Life sciences study design

All studies must disclose on these points even when the disclosure is negative.

Sample size	No calculations were done to determine sample size. Sample sizes were determined according to enable statistical analyses and on compared to previously published articles. All in vitro studies were repeated at least three times independently. The in vivo sample sizes were determined based on standards for animal studies and were comparable to previously published articles. For in vitro studies, sample size was 3-6, and for in vivo was 4-8 mice per group. The samples sizes were sufficient to detect meaningful biological differences for good reproducibility of the data.
Data exclusions	No data were excluded from the analysis
Replication	Experiments were repeated at least three independent experiments with similar results. All experiments were reproduced to reliably support conclusions stated in the manuscript.
Randomization	Animals were distributed randomly into different groups. Each specific treatment was administrated to animals according to established regimens. Same cells number were used for the experiments and all were well controlled. For in vitro studies, control and treated groups were divided from the same cell line or blood donors, so no randomization could be applied. For donor blood samples, healthy donors were randomly selected from our research team with ages ranging between 35-40. For aseptic loosening cases, patients were selected based on strict criteria recommended by our senior orthopedic doctors in our institution, including symptoms of aseptic loosening, X ray of hip, and no infection around the implant. So no randomization could be applied for selecting the human samples.
Blinding	Investigators were blinded when grouping and acquiring data. Investigators were blinded to group allocation during data analysis. Quantitative measurements were under control condition and performed by at least two different investigators to minimize the investigators interpretation during data collection.

Reporting for specific materials, systems and methods

We require information from authors about some types of materials, experimental systems and methods used in many studies. Here, indicate whether each material, system or method listed is relevant to your study. If you are not sure if a list item applies to your research, read the appropriate section before selecting a response.

Materials & experimental systems

n/a	Involved in the study	
[ ]	[x]	Antibodies
[ ]	[x]	Eukaryotic cell lines
[x]	[ ]	Palaeontology and archaeology
[ ]	[x]	Animals and other organisms
[ ]	[x]	Human research participants
[x]	[ ]	Clinical data
[x]	[ ]	Dual use research of concern

Methods

n/a	Involved in the study	
[x]	[ ]	ChIP-seq
[ ]	[x]	Flow cytometry
[x]	[ ]	MRI-based neuroimaging

Antibodies

Antibodies used

For In vivo depletion experiments: Injection dose: 150 µg
In VivoPlus Anti-mouse Ly6G (clone 1A8, Cat#: BP0075-1, BioX Cell, N Labombard Rd, Lebanon, USA)
In Vivo MAb rat IgG2 Isotype control (clone 2A3, Cat#: BE0089, BioX Cell, N Labombard Rd, Lebanon, USA)

Western blotting
Primary antibody
Anti-annexin A1 AnxA1 antibody (BL28553, 1:1000 dilution, Biolegend, San Diego, USA)
Anti-β-actin antibody (SP124, 1:2000 dilution, Abcam, UK)
Anti-total NFκB P65 (14G10A21, 1:1000 dilution, Biolegend San Diego, USA)
Anti-NFkB p65 (phospho Ser536) antibody (GTX133899, 1:1000 dilution, GeneTex, CA, USA)
Anti-phospho-NF-κB p105 (Ser932) antibody (18E6, #4806, 1:1000 dilution, Cell signaling technology, CST, MA, USA)
Anti-phospho-p38 MAPK (T180/Y182) antibody (D3F9, #4511, 1:1000 dilution, Cell signaling technology, CST, MA, USA)
Anti-phospho-RelB (Ser552) (D41B9, #5025, 1:1000 dilution, Cell signaling technology, CST, MA, USA)
Anti-PPARγ antibody (D8I3Y, #95128, 1:1000 dilution, Cell signaling technology, CST, MA, USA)

Secondary antibodies:
Anti-mouse HRP conjugated antibody (7076, 1:2000 dilution; Cell signaling technology, CST, MA, USA)
Anti-rabbit HRP conjugated antibody (7074P2, 1:2000 dilution; Cell signaling technology, CST, MA, USA)

For IHC
Rabbit anti-human Myeloperoxidase MPO antibody (JM10-58, 1:200 dilution, Novus Biologicals, USA)
Rabbit anti-Neutrophil Elastase antibody (ab68672, 1:200 dilution Abcam, Cambridge, UK)
Anti-human CD68 (KP1, clone PG-M1, M0876: 1:200 dilution, Dako Agilent, USA)
Anti-annexin A1 AnxA1 Rb antibody (GTX113329; 1:200 dilution, GeneTex, CA, USA)

The signals for IHC were amplified with Vectastain Elite ABC kit for detection of peroxidase (PK-6102 for mouse, PK-6103 for human; Vector Laboratories, Burlingame, USA)

Validation

All antibodies used have been validated by the manufactures. Protocols for immunostaining and Western Blotting were performed according to the manufacturer's instructions
Antibodies for In vivo depletion experiments
LY6G: https://bxcell.com/product/invivoplus-anti-m-ly-6g-2/
Control Ab: https://bxcell.com/product/rat-igg2a-isotype-control/

Antibodies for Western blotting
AnxA1 :https://www.biolegend.com/ja-jp/products/purified-anti-annexin-a1-antibody-17168
B actin: https://www.abcam.co.jp/beta-actin-antibody-sp124-ab115777.html
tP65: https://www.biolegend.com/ja-jp/products/purified-anti-nf-kappab-p65-antibody-8209
pP65: https://www.genetex.com/Product/Detail/NFkB-p65-phospho-Ser536-antibody/GTX133899
pP105: https://en.cellsignal.jp/products/primary-antibodies/phospho-nf-kb-p105-ser933-18e6-rabbit-mab/4806
pP38: https://en.cellsignal.jp/products/primary-antibodies/phospho-p38-mapk-thr180-tyr182-d3f9-xp-rabbit-mab/4511
pRelB: https://en.cellsignal.jp/products/primary-antibodies/phospho-relb-ser552-d41b9-xp-rabbit-mab/5025
PPARg: https://en.cellsignal.jp/products/primary-antibodies/pparg-d8i3y-mouse-mab/95128

Anti-mouse HRP: https://en.cellsignal.jp/products/secondary-antibodies/anti-mouse-igg-hrp-linked-antibody/7076?site-search-type=Products&N=4294956287&Ntt=secondary+antibodies&fromPage=plp
Anti-rabbit HRP: https://en.cellsignal.jp/products/secondary-antibodies/anti-rabbit-igg-hrp-linked-antibody/7074

Antibodies for IHC
MPO: https://www.novusbio.com/products/myeloperoxidase-mpo-antibody-jm10-58_nbp2-66964
Elastase: https://www.abcam.com/neutrophil-elastase-antibody-ab68672.html
CD68: https://www.agilent.com/store/en_US/Prod-M087601-2/M087601-2
AnxA1 : https://www.genetex.com/Product/Detail/Annexin-A1-antibody/GTX113329
Detection of peroxidase mouse: https://vectorlabs.com/products/abc-kits/vectastain-elite-abc-hrp-kit-mouse-igg
Detection of peroxidase mouse: https://vectorlabs.com/products/abc-kits/vectastain-elite-abc-hrp-kit-human-igg

Eukaryotic cell lines

Policy information about cell lines

Cell line source(s)	Human fetal osteoblasts (Cell Applications San Diego, CA, USA)<br>Human monocyte cell line THP1 (RIKEN, Saitama, Japan)<br>Human fibroblast-like synoviocytes (Cell Applications San Diego, CA, USA)
Authentication	Suppliers used morphology and PCR based approaches to confirm the identity of human cell lines and to rule out both intra- and interspecies contamination. Morphological features of the cell line were frequently checked in our laboratory.
Mycoplasma contamination	All cells were negative for mycoplasma.
Commonly misidentified lines (See ICLAC register)	No commonly misidentified cell line were employed in this study.

Animals and other organisms

Policy information about studies involving animals; ARRIVE guidelines recommended for reporting animal research

Laboratory animals	All mice were kept in SPF condition and were maintained in controlled temperature of 21–22 °C, humidity of 40 and 50% and a constant 12-h light/dark room during the experiments. SPF 8-week-old male Balb/c mice, male C57BL/6 mice (purchased from CLEA, Tokyo, Japan) and were AnxA1 KO used in this study.
Wild animals	No wild animals were used in this study.
Field-collected samples	This study did not involve samples collected from the field.
Ethics oversight	Procedures for animal experiments were performed in accordance with our approved protocols (nos. 17-0085 & 18-0171) by the Institute of Animal Care and Use Committee of the Hokkaido University Graduate School of Medicine.

Note that full information on the approval of the study protocol must also be provided in the manuscript.

Human research participants

Policy information about studies involving human research participants

Population characteristics	One male of 60-year old and two females of 54- and 59-year old undergoing revision of total hip arthroplasty<br>Hip cases hip osteoarthritis (female 64-year-old) undergoing primary hip arthroplasty
Recruitment	Three patients undergoing revision of total hip arthroplasty due to aseptic loosening. All clinical samples were obtained from Hokkaido University Hospital and were selected based on strict controlled criteria recommended by our senior orthopedic doctors in our institution, including symptoms of aseptic loosening, X ray of hip, and no infection around the implant. The cases were blindly selected from the listed patients undergoing primary or revision of total hip arthroplasty in the Hokkaido University Hospital to ensure blinding approach.
Ethics oversight	The research protocols for human samples were approved by the Research Ethics Review Committee of Hokkaido University Hospital (Approval ID: 016-0002), and informed consents were obtained from all donors.

Note that full information on the approval of the study protocol must also be provided in the manuscript.

Flow Cytometry

Plots

Confirm that:

- [x] The axis labels state the marker and fluorochrome used (e.g. CD4-FITC).
- [x] The axis scales are clearly visible. Include numbers along axes only for bottom left plot of group (a 'group' is an analysis of identical markers).
- [ ] All plots are contour plots with outliers or pseudocolor plots.
- [x] A numerical value for number of cells or percentage (with statistics) is provided.

Methodology

Sample preparation	Single cell suspensions from spleen were used for flow cytometry. Spleens were harvested on day 7 and splenocytes were separated and resuspended in ice-cold phosphate-buffered saline (PBS) containing 0.5% bovine serum albumin. Thereafter, the cells were stained with fluorescein isothiocyanate-labeled (FITC) anti-mouse monoclonal antibodies for Gr.1, CD11b, CD11c, CD38, or CD19 and with phycoerythrin (PE) for F4/80 or CD3 (BioLegend, CA, USA), and analyzed by flow cytometry (BD FACSCalibur, USA).

Instrument	BD FACSCalibur, USA
Software	BD FACSDiva software to collect the data
Cell population abundance	Splenocytes
Gating strategy	Cell debris and dead cells were excluded and the gate was selected for Gr.1+ cells

☒ Tick this box to confirm that a figure exemplifying the gating strategy is provided in the Supplementary Information.
